# Characterisation of the nicotianamine aminotransferase and deoxymugineic acid synthase genes essential to Strategy II iron uptake in bread wheat (*Triticum aestivum* L.)

**DOI:** 10.1371/journal.pone.0177061

**Published:** 2017-05-05

**Authors:** Jesse T. Beasley, Julien P. Bonneau, Alexander A. T. Johnson

**Affiliations:** School of BioSciences, The University of Melbourne, Melbourne, Victoria, Australia; Murdoch University, AUSTRALIA

## Abstract

Iron (Fe) uptake in graminaceous plant species occurs via the release and uptake of Fe-chelating compounds known as mugineic acid family phytosiderophores (MAs). In the MAs biosynthetic pathway, nicotianamine aminotransferase (NAAT) and deoxymugineic acid synthase (DMAS) enzymes catalyse the formation of 2’-deoxymugineic acid (DMA) from nicotianamine (NA). Here we describe the identification and characterisation of six *TaNAAT* and three *TaDMAS1* genes in bread wheat (*Triticum aestivum* L.). The coding sequences of all six *TaNAAT* homeologs consist of seven exons with ≥88.0% nucleotide sequence identity and most sequence variation present in the first exon. The coding sequences of the three *TaDMAS1* homeologs consist of three exons with ≥97.8% nucleotide sequence identity. Phylogenetic analysis revealed that the TaNAAT and TaDMAS1 proteins are most closely related to the HvNAAT and HvDMAS1 proteins of barley and that there are two distinct groups of TaNAAT proteins—TaNAAT1 and TaNAAT2 –that correspond to the HvNAATA and HvNAATB proteins, respectively. Quantitative reverse transcription-PCR analysis revealed that the *TaNAAT2* genes are expressed at highest levels in anther tissues whilst the *TaNAAT1* and *TaDMAS1* genes are expressed at highest levels in root tissues of bread wheat. Furthermore, the *TaNAAT1*, *TaNAAT2* and *TaDMAS1* genes were differentially regulated by plant Fe status and their expression was significantly upregulated in root tissues from day five onwards during a seven-day Fe deficiency treatment. The identification and characterization of the *TaNAAT1*, *TaNAAT2* and *TaDMAS1* genes provides a valuable genetic resource for improving bread wheat growth on Fe deficient soils and enhancing grain Fe nutrition.

## Introduction

Graminaceous plant species acquire Fe from the rhizosphere through the release and uptake of Fe-chelating mugineic acid phytosiderophores (MAs), a process known as Strategy II Fe uptake. Despite the abundance of Fe in soils, its tendency to form insoluble ferric Fe [Fe(III)] precipitates under aerobic conditions at neutral (~7) pH levels often renders this essential micronutrient unavailable for uptake by living organisms [1]. Chelation of Fe(III) by MAs greatly increases metal solubility and enables uptake of Fe(III)-MAs complexes into plant roots. The quantity of MAs secreted by a graminaceous plant species correlates positively with the degree of tolerance to soils with low Fe bioavailability. For example, rice (*Oryza sativa* L.) and maize (*Zea mays* L.) secrete low amounts of MAs and grow poorly in Fe limiting environments such as calcareous soil with pH > 8 [2, 3]. By contrast, barley (*Hordeum vulgare* L.) and wheat (*Triticum aestivum* L.) secrete large amounts of MAs and demonstrate greatly increased tolerance to Fe limiting soils [2]. Barley secretes a range of MA species including mugineic acid (MA), 3-epihydroxymugineic acid (epiHMA), 3-epihydroxy-2’-deoxymugineic acid (epiHDMA) and 2’-deoxymugineic acid (DMA) whereas bread wheat only secretes DMA [4–6].

Synthesis of DMA occurs from nicotianamine (NA) via a 3”-oxo intermediate using nicotianamine aminotransferase (NAAT) and deoxymugineic acid synthase (DMAS) enzymes. While NA is biosynthesized in all plant species, the transamination of NA by NAAT is a reaction unique to graminaceous plants and one that represents the first step towards strategy II Fe uptake [7]. Aminotransferase enzymes similar to NAAT, such as tyrosine aminotransferase (TAT), are active in non-graminaceous plant species and function in the biosynthesis of compounds such as rosmarinic acid and benzylisoquinoline alkaloids that are not involved in plant Fe uptake [8, 9]. Reduction of the 3”-oxo intermediate produced by NAAT is catalysed by DMAS, an enzyme belonging to the large enzyme superfamily of aldo-keto reductases [10].

The genes encoding NAAT and DMAS enzymes have been characterised in several graminaceous monocots including rice and barley [11–13]. Rice has six NAAT genes that comprise the *OsNAAT* gene family and one member, *OsNAAT1*, shows highest expression levels in root tissues and significant upregulation in response to Fe deficiency [12, 14]. Expression of the other five *OsNAAT* genes does not change in response to Fe deficiency and the function(s) of these genes remains unknown [12]. Barley contains two NAAT genes, *HvNAATA* and *HvNAATB*, both of which show ≥74% sequence homology to the rice *OsNAAT1* gene [12, 13, 15]. As with the rice gene *OsNAAT1*, expression of the *HvNAAT* genes is root-specific and upregulated in response to Fe deficiency [13]. The *OsDMAS1* and *HvDMAS1* genes are responsible for DMA biosynthesis in rice and barley, respectively [11]. Expression of the *HvDMAS1* gene is induced in barley root tissues exposed to Fe deficient conditions [16]. By contrast, expression of *OsDMAS1* is specific to rice root tissues under conditions of Fe sufficiency and is induced in both root and shoot tissues under Fe deficiency. These expression patterns indicate that DMA plays important roles in maintaining Fe homeostasis within graminaceous plant tissues in addition to its role in acquiring Fe from soil [11].

Wheat accounts for over 30% of global cereal production and is cultivated on more land than any other crop [17]. When grown under conditions of Fe deficiency, wheat plants exhibit leaf chlorosis and a reduction in yield [18, 19]. Given that an estimated 500 million hectares of the world’s soils are alkaline, novel strategies to increase wheat tolerance to Fe deficiency are needed to maximize production on these soil types [17]. As a consequence of the large differences in genomic complexity between rice (diploid, 2n = 24), barley (diploid, 2n = 14) and bread wheat (hexaploid, 2n = 6x = 42), many gene families that are well characterized in rice and barley have not been reported in bread wheat. The recent identification of 21 nicotianamine synthase (NAS) genes in bread wheat, the largest NAS gene family reported to date, highlights the genomic complexity of Fe homeostasis genes in polyploid cereal species such as wheat [20]. Although partial sequences of several NAAT and DMAS genes have been identified in bread wheat, a comprehensive characterization of these gene families has not been performed [11, 16]. Recent biochemical studies identifying NA and/or DMA as the predominant chelators of Fe in wheat white flour indicate that full characterization of these gene families could enable genetic strategies to improve Fe nutrition of this staple food [21, 22]. In this paper we describe the bread wheat *TaNAAT* (orthologous to *OsNAAT1*, *HvNAATA* and *HvNAATB*) and *TaDMAS1* (orthologous to *OsDMAS1* and *HvDMAS1*) gene families in terms of chromosomal location, phylogenetic relationship and expression in various tissues as well as under conditions of Fe deficiency. The results greatly expand our knowledge of Strategy II Fe uptake genetics in bread wheat and could lead to the development of wheat cultivars with enhanced tolerance to Fe limiting environments as well as improved grain Fe nutrition.

## Results and discussion

### Six *TaNAAT* genes are located on chromosomal group 1 and three *TaDMAS1* genes are located on chromosomal group 4

Six *TaNAAT* and three *TaDMAS1* genes were identified within the bread wheat genome (Table 1). The three *TaNAAT1* genes and the three *TaNAAT2* genes are located on chromosomal group 1 and the three *TaDMAS1* genes are located on chromosomal group 4. All nine of the genes are located on the long arm of their respective chromosomal groups with the exception of *TaDMAS1-A* which is located on the short arm of chromosome 4A due to a known pericentric inversion between the long and short chromosome arms [23]. The *TaNAAT1*, *TaNAAT2* and *TaDMAS1* genes share a syntenic relationship with the orthologous barley *HvNAATA*, *HvNAATB* and *HvDMAS1* genes that are located on barley chromosomes 1H and 4H, respectively [24].

**Table 1:** The *TaNAAT1*, *TaNAAT2* and *TaDMAS* genes of bread wheat.

Gene name	Chromosome location	IWGSC contig	MIPS database gene annotation	GenBank accession no.	Gene length (bp)
TaNAAT1-A*	1AL	ctg3907095	Traes_1AL_9D6B86169	KX348554	3236
*TaNAAT1-B*	1BL	ctg3795417	Traes_1BL_9567F31C9.1	KX348555	3114
*TaNAAT1-D*	1DL	ctg2261959	Traes_1DL_7B4106561	KX348556	3921
TaNAAT2-A*	1AL	ctg3883773	Traes_1AL_BCD7C5B8B	KX348557	3036
TaNAAT2-B*	1BL	ctg3814337	Traes_1BL_D8276D3DB	KX348558	3102
*TaNAAT2-D*	1DL	ctg604261	Traes_1DL_4D8CFB738.2	KX348559	3194
TaDMAS1-A*	4AS	ctg5955230	Traes_4AS_887399584	KX348551	1794
TaDMAS1-B*	4BL	ctg7035734	Traes_4BL_FAB8CACD6	KX348552	1831
*TaDMAS1-D*	4DL	ctg14416645	Traes_4DL_01BE8F7DE	KX348553	1772

IWGSC, International Wheat Genome Sequencing Consortium; MIPS, Munich Information Centre for Protein Sequences.

* correspond to the *TaNAAT1*, *TaNAAT2* and *TaDMAS1* genes, respectively, described in [16]

**Fig 1:**
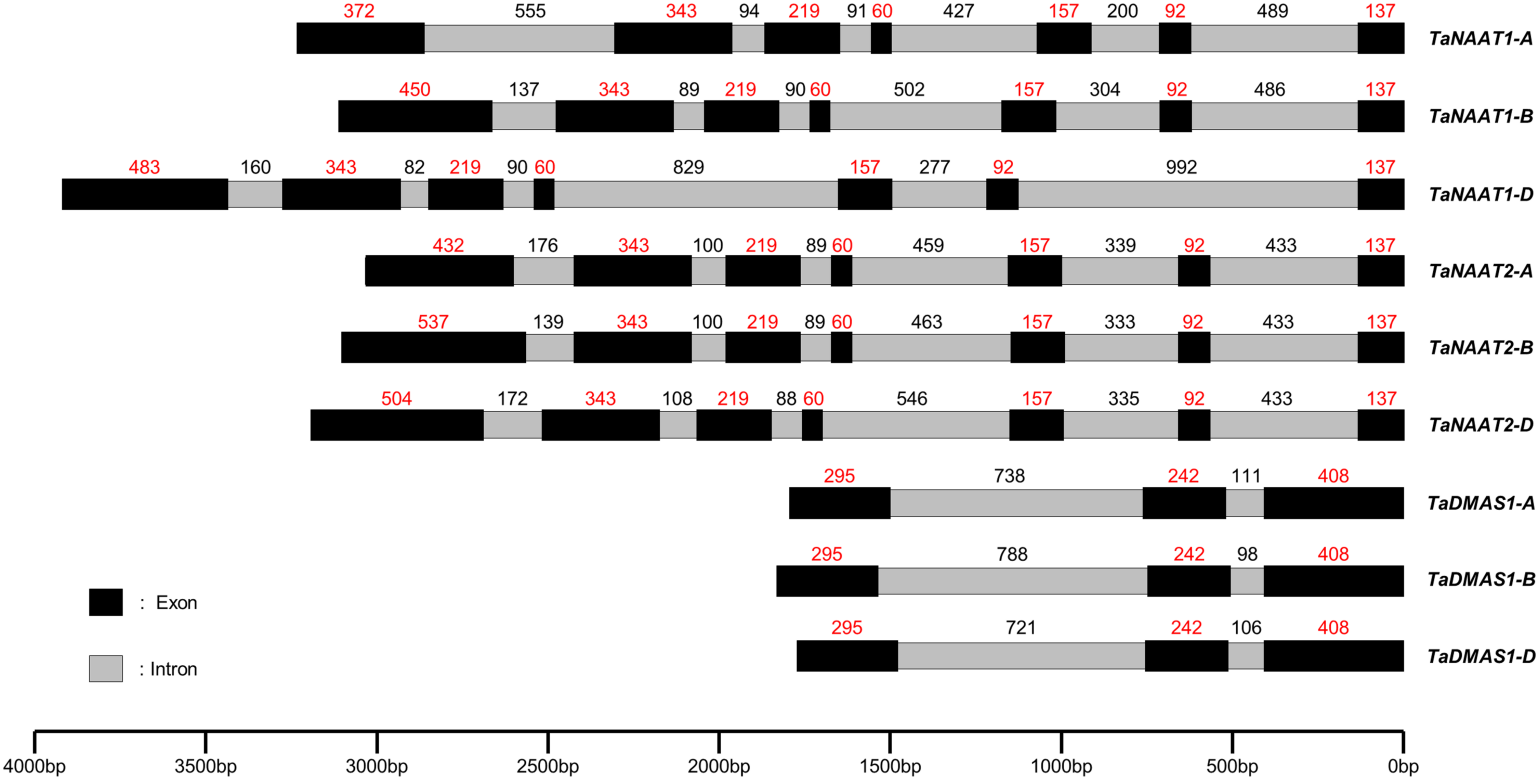
Sequence structure and length of the *TaNAAT1*, *TaNAAT2* and *TaDMAS1* genes in bread wheat cv. Gladius. The figure shows exons and introns within the *TaNAAT1*, *TaNAAT2* and *TaDMAS1* genes depicted in black and grey, respectively. Exon and intron length (bp) is indicated in red and black, respectively. Variation in sequence length was observed in most intronic regions and in the first exon for each of the *TaNAAT* genes.

The genomic sequences of the six *TaNAAT* genes range in size from 3000–3900 bp due to differences in intron length as well as length of the first exon (Fig 1). The coding sequences of the six *TaNAAT* genes are comprised of seven exons and range from 1380–1491 bp for the three *TaNAAT1* genes and 1440–1545 bp for the three *TaNAAT2* genes. By comparison, the coding sequences of the orthologous *HvNAATA* and *HvNAATB* genes in barley are 1386 bp and 1653 bp in length, respectively [13]. Variation in size of the *TaNAAT* coding sequences is due solely to variable length of the first exon; the lengths of the remaining six exons are identical across all six *TaNAAT* genes (Fig 1). The nucleotide sequence identity between the coding sequences of the three *TaNAAT1* genes and the three *TaNAAT2* genes is ≥88.8% with the first exon and ≥95.7% without the first exon (S1 Table). Variability in *TaNAAT* gene coding sequences between cultivars Gladius and Chinese Spring was only detected for the *TaNAAT1* and *TaNAAT2* genes located on subgenome B. The coding sequence of the *TaNAAT1-B* gene in cv. Gladius was found to be 30 bp shorter than that of cv. Chinese Spring due to a deletion in the first exon. Additionally, 10 single nucleotide polymorphisms (SNPs) were detected in the *TaNAAT2-B* coding sequences between the two cultivars. The detection of significant sequence length variation between homeologous members of the *TaNAAT1* and *TaNAAT2* gene families supports the hypothesis of an accelerated evolution of homeologous genes in the polyploid bread wheat genome as a result of genetic redundancy and therefore greater tolerance for sequence alterations [25]. Variability in first exon sequence length between the homeologous genes may in fact represent early stages of gene sub-functionalization and neo-functionalization in the *TaNAAT* genes [26]. Despite the sequence length variation, expression of all six *TaNAAT* genes was detected in both cDNA libraries utilized in this study with similar spatial and temporal expression patterns between the three *TaNAAT1* genes and the three *TaNAAT2* genes. The genomic sequences of the three *TaDMAS1* genes range in size from 1770–1830 bp due to variable intron sequence length (Fig 1). The coding sequences of the three *TaDMAS1* genes, in contrast to the six *TaNAAT* genes, do not vary in size and are comprised of three exons totalling 945 bp and share ≥97.8% nucleotide sequence identity (S2 Table). The open reading frame of the orthologous *HvDMAS1* gene in barley is also 945 bp in length [11].

**Fig 2:**
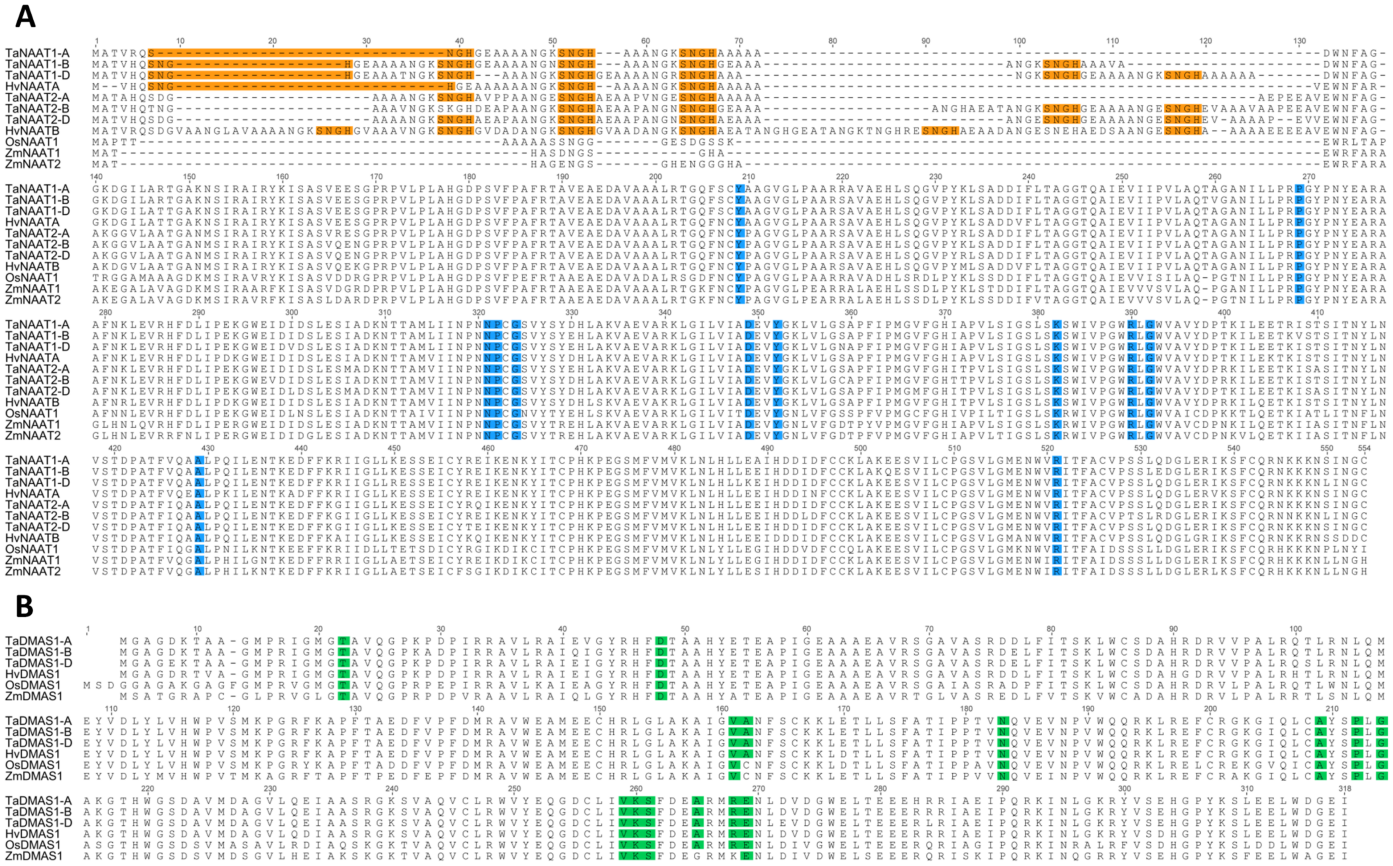
Amino acid alignment of the NAAT and DMAS1 proteins of rice (Os), barley (Hv), maize (Zm) and wheat (Ta) identifies conserved regions and motifs. (A) Blue shading corresponds to residues critical to aminotransferase function. The SNGH repeats in the N terminal region of NAAT proteins are shaded in orange (S = serine, N = asparagine, G = glycine and H = histidine). (B) Green shading corresponds to residues within the nicotinamide adenine dinucleotide phosphate (NADPH) binding domain of DMAS1 proteins.

The amino acid sequences of the six TaNAAT and three TaDMAS1 proteins contain conserved residues that are characteristic of their enzyme superfamilies (Fig 2). All TaNAAT proteins contain a Lys residue (K393) thought to be critical for pyridoxal phosphate binding and enzyme activation [13]. In addition, the six TaNAAT proteins contain between three and six repeated SNGH amino acid motifs in the N terminal region (Fig 2A). Repeated SNGH amino acid residues of unknown function have also been identified in the two NAAT proteins of barley, suggesting that these sequences are a characteristic feature of NAAT proteins belonging to the *Triticeae* species [13]. The TaNAAT1-B protein sequence contains one less SNGH repeat in cv. Gladius compared to cv. Chinese Spring, and this difference between the two cultivars provides an opportunity to assess essentiality of the SNGH motif to NAAT protein function in future studies. All three TaDMAS1 proteins contain putative nicotinamide adenine dinucleotide phosphate (NADPH) binding residues that are also present in rice and barley DMAS proteins [11] (Fig 2B).

### The TaNAAT and TaDMAS1 proteins share a common origin with HvNAAT and HvDMAS1 proteins

**Fig 3:**
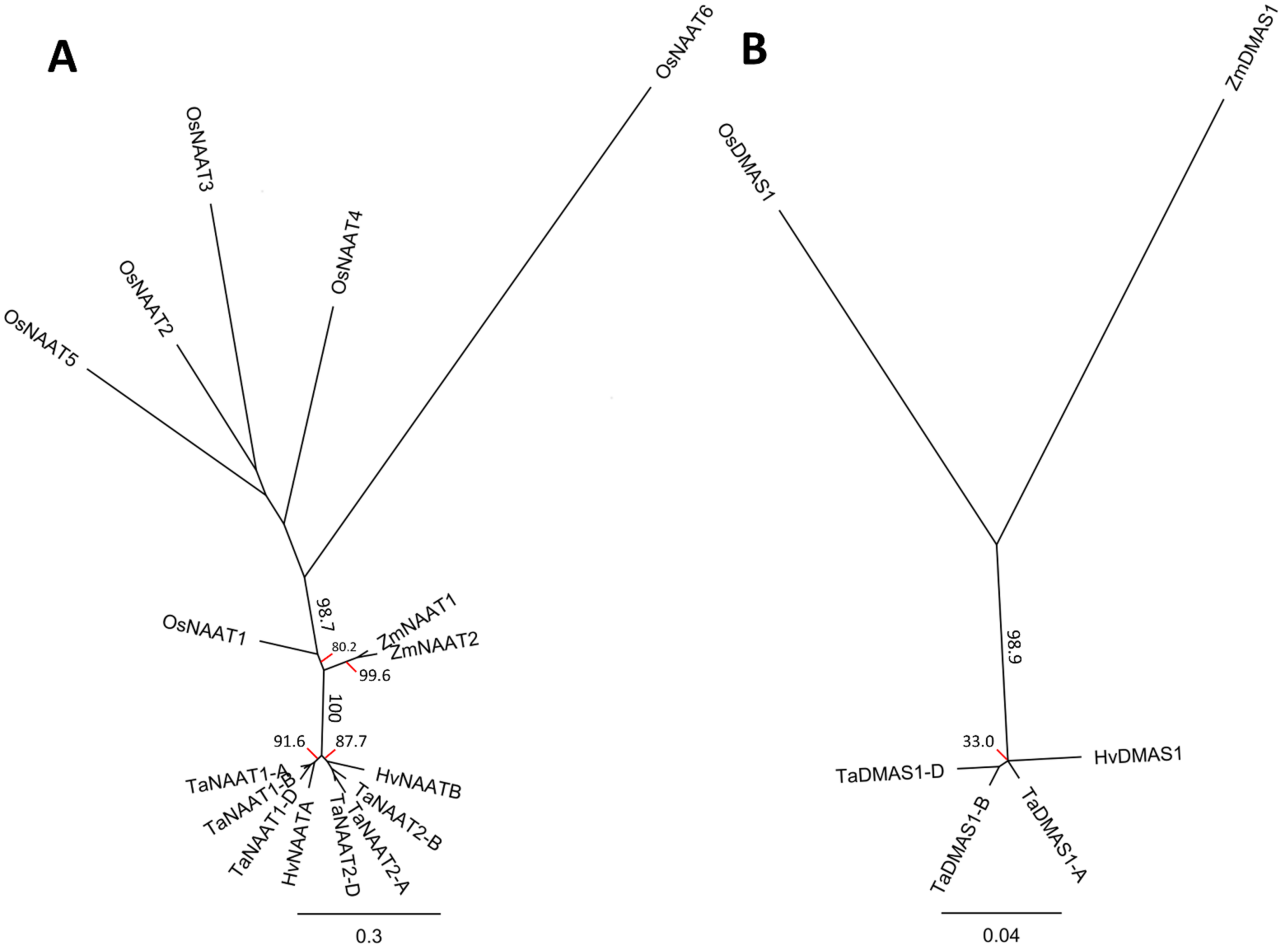
Phylogenetic trees of the NAAT and DMAS1 proteins of wheat, rice, barley and maize demonstrate that the wheat and barley proteins group separately from those of rice and maize. Unrooted phylogenetic trees of (**A**) NAAT proteins from wheat (TaNAAT), rice (OsNAAT), maize (ZmNAAT), and barley (HvNAAT) and (**B**) DMAS1 proteins from wheat (TaDMAS1), rice (OsDMAS1), maize (ZmDMAS1), and barley (HvDMAS1). The scale bar represents evolutionary distance in substitutions per base and the numbers reflect bootstrap percentage.

Phylogenetic analyses of the NAAT and DMAS protein sequences from rice, maize, barley and wheat revealed that the wheat TaNAAT and TaDMAS1 proteins are most similar to those of barley (Fig 3). All TaNAAT proteins in bread wheat grouped with the barley HvNAATA and HvNAATB proteins (bootstrap proportion of 100%) and separately from the NAAT proteins of rice and maize (Fig 3A). The six TaNAAT and two HvNAAT proteins separated into two subgroups based on a bootstrap proportion of 91.6% for TaNAAT1/HvNAATA and 87.7% for TaNAAT2/HvNAATB (Fig 3A). It was originally hypothesised that the barley HvNAATA and HvNAATB proteins were isozymes encoded by one NAAT gene in barley [15]. However, the presence of two NAAT subgroups in wheat and barley indicates that in fact a NAAT gene duplication event occurred in the diploid wheat-barley ancestor after the divergence of these two species from other graminaceous monocots. Phylogenetic analysis of the DMAS1 protein sequences of rice, barley, maize and wheat produced a similar relationship, with the HvDMAS1 and TaDMAS1 proteins grouping separately from the DMAS1 proteins of rice and maize (Fig 3B).

### The *TaNAAT1* and *TaNAAT2* genes are highly expressed in root and anther tissues while the *TaDMAS1* genes are broadly expressed across different tissues and developmental stages

**Fig 4:**
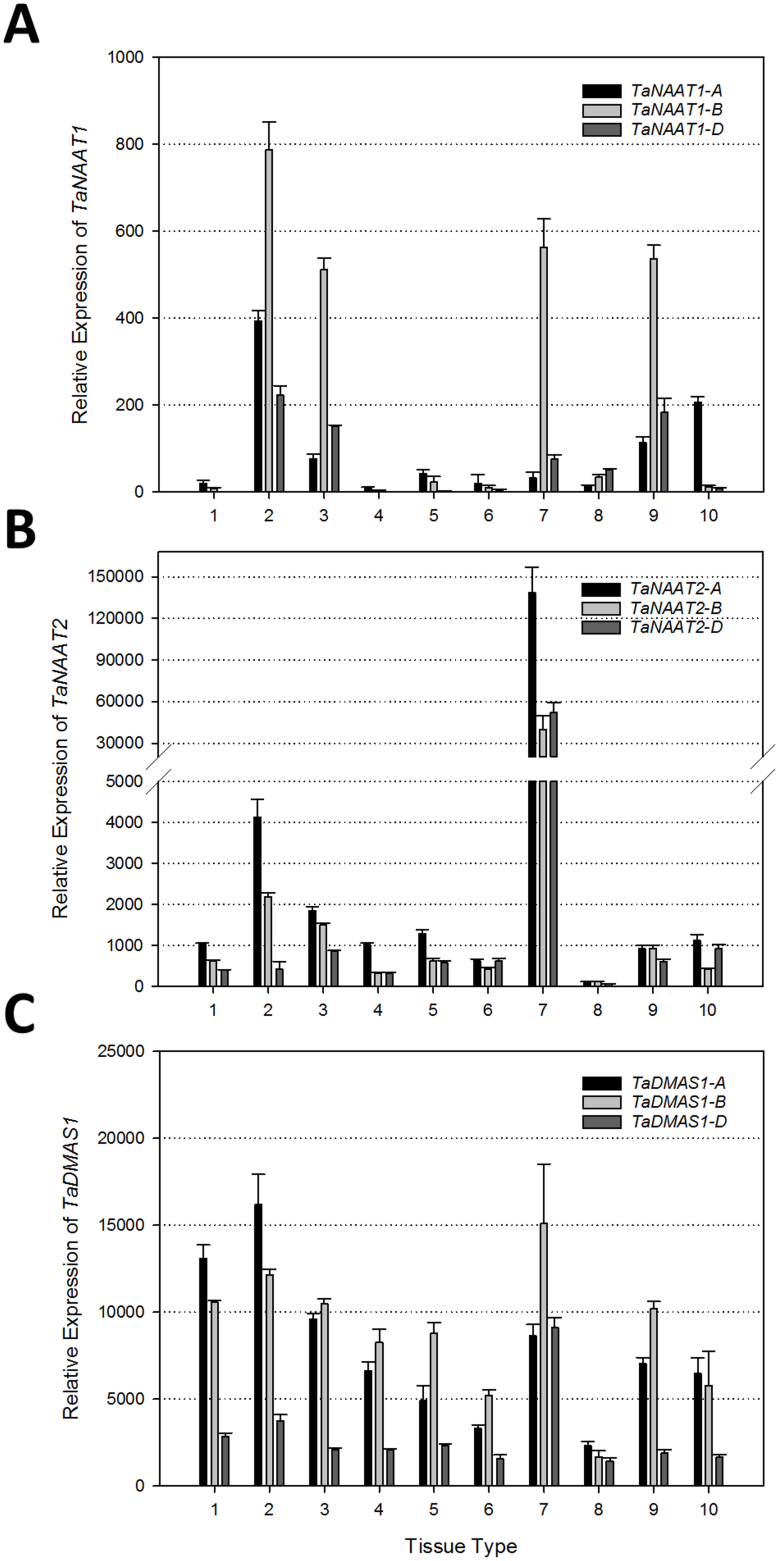
Relative expression of the *TaNAAT1*, *TaNAAT2* and *TaDMAS1* genes in 10 different tissues and developmental stages of bread wheat cv. Chinese Spring. Relative expression of (**A**) *TaNAAT1*, (**B**) *TaNAAT2* and (**C**) *TaDMAS1* genes is provided in: (1) mesocotyl and (2) embryonic root; (3) seedling root, (4) crown and (5) seedling leaf; (6) mature bracts, (7) anthers and (8) pistil; (9) caryopsis—3/5 DAP and (10) embryo—22 DAP. Units of the y-axis indicate copies of mRNA per μl of cDNA. Error bars indicate standard deviation of the mean of three technical replicates derived from a bulk of 3 biological replicates.

Quantitative reverse transcription-PCR analysis of a range of bread wheat tissues and developmental stages of cv. Chinese Spring revealed distinctive expression profiles in root and anther tissues for the *TaNAAT1*, *TaNAAT2* and *TaDMAS1* genes (Fig 4A–4C). Expression levels were highest for the *TaNAAT1* homeolog found on subgenome B (*TaNAAT1-B*), the *TaNAAT2* homeolog found on subgenome A (*TaNAAT2-A*) and the *TaDMAS1* homeolog found on subgenome A (*TaDMAS1-A*). These expression trends were observed in both the Chinese Spring and Gladius cultivars utilized in this study (S1 Fig). Similar differential expression of homeologous genes has been documented in response to heat, drought and pathogen stresses in bread wheat [27–29].

Expression of the three *TaNAAT1* genes was approximately 100-fold lower than that of the *TaNAAT2* and *TaDMAS1* homeologs and most *TaNAAT1* gene expression was detected in root tissues at the embryonic and seedling stages as well as in the caryopsis (Fig 4A). Expression of the three *TaNAAT2* genes was approximately 100 to 250-fold higher in the anthers relative to all other organs examined (Fig 4B). In addition to anthers, the three *TaNAAT2* genes were also highly expressed in root tissues at the embryonic and seedling stage. Both groups of *TaNAAT* homeologous genes had low levels of expression in the mesocotyl, crown, leaf, bracts and pistil tissues (Fig 4A and 4B). By contrast, the three *TaDMAS1* genes were expressed at higher levels than both groups of *TaNAAT1* and *TaNAAT2* genes and across all tissue types (with the exception of the very high *TaNAAT2* expression levels in anthers). The three *TaDMAS1* genes were most highly expressed in embryonic mesocotyl and root tissues, seedling root tissues and in the anther (Fig 4C).

These results indicate that DMA plays critical roles during germination and early seedling growth stages of bread wheat by enabling Fe uptake from the rhizosphere and maintaining metal homeostasis. Furthermore, the detection of high *TaDMAS1* gene expression across many different tissue types and developmental stages of bread wheat suggests that DMA participates in long distance transport and grain loading of metal micronutrients; roles that have also been identified for DMA in rice [11, 21, 22, 30]. The finding that the three *TaNAAT2* genes were most highly expressed in anther tissues is an interesting result that has not been reported for other NAAT genes (Fig 4B). Interestingly, our search of the Affymetrix 22K Barley1 GeneChip showed that the barley orthologue of the three *TaNAAT2* homeologs, *HvNAATB*, also has highest expression levels in anther tissue [31–33]. A specific member of the NAS gene family of bread wheat, *TaNAS7-A2*, was recently reported to have 100-fold higher expression levels in anther tissue relative to all other wheat tissues examined [20]. Taken together, these results suggest that high activity of the NA and DMA biosynthetic pathway is required for proper anther development in bread wheat and barley. Previous studies in Arabidopsis, tobacco and rice have also identified NA and DMA as having critical roles in pollen function, anther development and reproduction [7, 34, 35].

Analysis of the 1kb promoter region of the *TaNAAT1* and *TaNAAT2* genes revealed variable positioning of the TATA-box (S2 Fig). Furthermore, multiple *cis*-acting regulatory elements that are known to confer pollen- and anther-specific expression in rice, tomato (*Solanum lycopersicum*) and tobacco were located in close proximity to, and downstream of, the *TaNAAT2* TATA-box [36–40]. These elements may contribute to the high level of *TaNAAT2* gene expression that we observed within the anther tissues of bread wheat. Interestingly, the *TaDMAS1* genes did not contain a TATA-box within the 1kb promoter region suggesting that these genes may be TATA-less (S2 Fig).

### Expression levels of the *TaNAAT1*, *TaNAAT2* and *TaDMAS1* genes are temporally regulated in wheat roots to enable rapid DMA biosynthesis in response to Fe deficiency and avoid NA depletion

**Fig 5:**
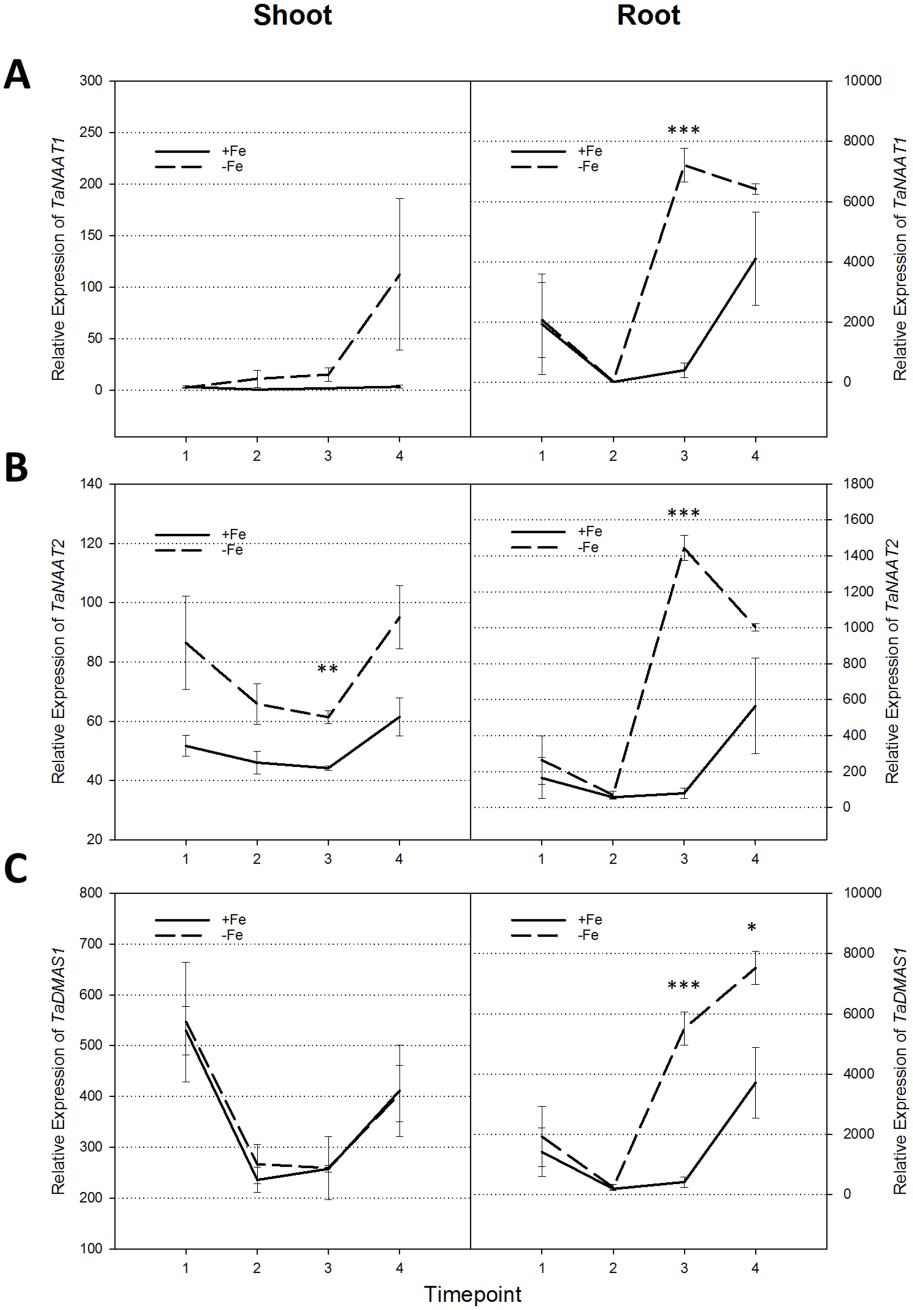
Relative expression of the *TaNAAT1*, *TaNAAT2* and *TaDMAS1* genes in shoot and root tissues of bread wheat cv. Gladius under iron-sufficient/deficient conditions. Averaged relative expression of the (**A**) *TaNAAT1*, (**B**) *TaNAAT2* and (**C**) *TaDMAS1* genes is presented at four time points of the 7 day treatment: days 0 (experiment start), 1, 5 and 7 of Fe-sufficient (+Fe, solid line) or Fe-deficient (-Fe, dashed line) conditions. Units of the y-axis indicate copies of mRNA per μl of cDNA. The error bars indicate standard error of the mean of three biological replicates for each of three genes (n = 9). Asterisks indicate significant differences for the effect of condition (+Fe and −Fe) at each time point (two-sample Student’s t-test assuming equal variance; * = p value ≤0.05; ** = p value ≤0.01; *** = p value ≤0.001).

The *TaNAAT1*, *TaNAAT2* and *TaDMAS1* genes showed significantly up-regulated expression in root tissues at day 5 of the Fe deficiency treatment (Fig 5A–5C). In barley, a similar increase in expression of the *HvNAAT* and *HvDMAS1* genes occurs in response to Fe deficiency [11, 13, 41]. In the shoot tissues, only the *TaNAAT2* genes showed a small increase in expression in response to Fe deficiency (Fig 5B), indicating that bread wheat (like barley) responds to Fe deficiency by increasing DMA biosynthesis predominately in root tissues. By contrast, the *OsNAAT1* and *OsDMAS1* genes of rice are significantly up-regulated in both root and shoot tissues in response to Fe deficiency [11, 12]. The 1kb promoter regions of the *TaNAAT1*, *TaNAAT2* and *TaDMAS1* genes all contain the *cis*-acting regulatory elements IDE1 and IDE2-like (S2 Fig). These elements have been identified in the promoters of several iron deficiency-responsive genes in *Arabidopsis*, rice and barley and likely contribute to the observed induction of *TaNAAT1*, *TaNAAT2* and *TaDMAS1* gene expression in root tissues of bread wheat under Fe deficiency [42].

Expression levels of all but one of the six *TaNAAT* genes decreased from day 5 onwards in bread wheat root tissues in response to Fe deficiency (Fig 5A and 5B). Only a single *TaNAAT* gene, *TaNAAT2-A*, remained significantly up-regulated (p ≤ 0.05) at day 7 (S1 Fig). By contrast, all three of the *TaDMAS1* genes were significantly up-regulated at days 5 and 7 in root tissues under Fe deficiency (Fig 5C). These results suggest that root-specific induction of *TaNAAT1* and *TaNAAT2* gene expression occurs early in the bread wheat Fe deficiency response to provide adequate amounts of the 3”-oxo intermediate substrate for DMA biosynthesis by TaDMAS1 enzymes (Fig 5A–5C). Subsequent down-regulation of most of the *TaNAAT* genes from day 5 onwards likely helps the plant to avoid depletion of internal NA reserves.

It is worth mentioning that DMA secretion increases not only under Fe deficiency, but also Zn deficiency, in wheat, barley and rice [43–46]. In future studies it could be useful to investigate expression of the *TaNAAT1*, *TaNAAT2* and *TaDMAS1* genes in response to Zn deficiency to determine if similar temporal regulation of the genes exists under this condition.

### The *TaNAAT1* and *TaNAAT2* genes function in different aspects of Fe homeostasis

Under conditions of Fe deficiency, the three *TaNAAT1* genes were expressed at higher levels than the three *TaNAAT2* genes in root tissues (Fig 5 and S1 Fig). Under conditions of Fe sufficiency, however, the three *TaNAAT2* genes were expressed at higher levels than the three *TaNAAT1* genes across a broad range of bread wheat tissues including very high expression levels in anthers (Fig 4A and 4B). These results suggest that the three *TaNAAT1* genes are mainly responsible for increased root DMA biosynthesis in response to low Fe availability while the three *TaNAAT2* genes play an important role in maintaining DMA biosynthesis in a wide range of organs under Fe sufficiency. The barley orthologues of the bread wheat *TaNAAT1* and *TaNAAT2* genes, *HvNAATA* and *HvNAATB*, respectively, show similar differences in expression pattern and apparent function. Induction of the *HvNAATA* gene is greater than that of *HvNAATB* in barley root tissues under conditions of Fe deficiency [13, 15]. The HvNAATA enzyme has also been shown to have greater affinity for NA compared to the HvNAATB enzyme, an important factor under conditions of Fe deficiency where NA levels are lowered and a fact that likely explains the preferential induction of the *HvNAATA* gene under Fe deficiency [15]. By contrast, the *HvNAATB* gene is expressed in barley leaf tissues during senescence-related Fe remobilisation as well as in grain transfer cells; *HvNAATA* expression is undetectable in both of these tissue types [47, 48].

### Applications to plant breeding

The identification and characterization of the *TaNAAT1*, *TaNAAT2* and *TaDMAS1* genes of bread wheat provides a novel genetic resource for improving bread wheat growth and nutrition via conventional breeding and biotechnology. Examination of allelic diversity in the *TaNAAT1*, *TaNAAT2* and *TaDMAS1* genes in future work could lead to the identification of novel gene variants with agronomically useful expression patterns. Ectopic expression of barley *HvNAAT* genes in rice using endogenous promoters resulted in transgenic rice plants with 1.8-fold increased DMA secretion under Fe deficient growth conditions and a 4-fold increase in yield [49]. Similar strategies to alter the expression of the *TaNAAT1* or *TaDMAS1* genes, or the identification of novel variants with altered expression levels, could increase the growth and yield of bread wheat on alkaline soils where Fe and other micronutrient metals are highly limiting. Alternatively, endosperm-specific overexpression of specific *TaNAAT2* or *TaDMAS1* genes that are highly expressed in anther tissues could increase grain Fe storage capacity and lead to the production of biofortified wheat varieties with higher grain Fe contents. Finally, the results of this study demonstrate that the *TaNAAT1*, *TaNAAT2* and *TaDMAS1* genes are similar in both sequence and expression pattern to orthologous genes in barley and that these gene families may be evolving to take on unique roles in the hexaploid bread wheat genome.

## Materials and methods

### Identification of *TaNAAT1*, *TaNAAT2* and *TaDMAS1* genes

The coding sequences of *HvNAATA*, *HvNAATB* and *HvDMAS1* genes (S3 Table) were used as BLAST queries against the International Wheat Genome Sequencing Consortium (IWGSC) databases (IWGSC, 2014—http://www.wheatgenome.org) to identify sequences encoding putative *TaNAAT1*, *TaNAAT2* and *TaDMAS1* genes. The 1kb promoter regions were also identified for the *TaNAAT1-A* (contig_3507095), *TaNAAT1-D* (contig_2269183), *TaNAAT2-A* (contig_3876687), *TaNAAT2-D* (contig_2204013), *TaDMAS1-A* (contig_6010427), *TaDMAS1-B* (contig_7035734) and *TaDMAS1-D* (contig_14416645) genes. Matching sequences from the IWGSC databases were then annotated using FGENESH (http://www.softberry.com) software [50]. For the *TaNAAT1-B*, *TaNAAT1-D*, *TaNAAT2-A*, *TaNAAT2-B* and *TaNAAT2-D* genes which did not return full genomic sequences from IWGSC, we further searched the *Triticum aestivum* cv. Chinese Spring 5x genome database (http://www.cerealsdb.uk.net) as well as databases containing whole-genome assemblies of *Triticum durum* (AABB), *Triticum monococcum* (AA) and *Aegilops tauschii* (DD) (https://wheat-urgi.versailles.inra.fr/Seq-Repository/Assemblies). These results were used to assemble *in silico* full genomic sequences for *TaNAAT1*, *TaNAAT2* and *TaDMAS1* genes within cv. Chinese Spring. Each *TaNAAT1* (*TaNAAT1-A*/*TaNAAT1-B*/*TaNAAT1-D*) *TaNAAT2* (*TaNAAT2-A*/*TaNAAT2-B*/*TaNAAT2-D*) and *TaDMAS1* (*TaDMAS1-A*/*TaDMAS1-B*/*TaDMAS1-D*) gene was named according to homeologous grouping and subgenome localisation using the recommended rules for gene symbolisation in bread wheat (http://wheat.pw.usda.gov/ggpages/wgc/98/Intro.htm). Homeologous groups were defined on the basis of an empirical nucleotide identity threshold of ≥88.8% between coding sequences of individual genes (S1 and S2 Tables). The classification of the *TaDMAS1-A* gene took into account the known pericentromeric inversion of wheat chromosome 4AS-4AL [23].

Predictions of coding sequences (CDS) and protein sequences were obtained for each of the *TaNAAT1*, *TaNAAT2* and *TaDMAS1* genes using the gene prediction software FGENESH. The coding sequences of all *TaNAAT1*, *TaNAAT2* and *TaDMAS1* genes were then validated using BLASTN searches to the TGACv1 genome assembly (http://pre.plants.ensembl.org/Triticum_aestivum/Info/Index), all of which returned a single TGAC scaffold with ≥99% identity. Annotation of *cis*-acting elements within the 1kb promoter regions of the *TaNAAT* and *TaDMAS1* genes included: PB Core (CCAC), the enhancer elements *LAT52* (TGTGG) and *LAT56* (TGTGA), the pollen-specific enhancer element *LAT52* (AGAAA), and the *g10* late pollen gene element (GTGA) [36–40]. The *cis*-acting IDE1 (ATCAAGCATGCTTCTTGC) and IDE2-like (TTGAACGGCAAGTTTCACGCTGTCACT) elements were identified as previously described with a minimum of 51% sequence identity [51]. Alignment of CDS (CLUSTALW) and protein sequences (ClustalW with cost matrix BLOSUM, gap open cost 10), and annotation of 1kb promoter regions, was performed in Geneious Pro 8.1.7 software (Biomatters, http://www.geneious.com).

### Sequencing of the *TaNAAT1*, *TaNAAT2* and *TaDMAS1* genes

A combination of 3–5 pairs of primers were used for complete coverage of *TaNAAT1*, *TaNAAT2* and *TaDMAS1* genomic sequences in cvs. Chinese Spring and Gladius (Table 1). Subgenome specificity of primer pairs was verified using cv. Chinese Spring nulli-tetrasomic DNA [52]. All PCR reactions were performed in a total volume of 20 μl using MyTaq^™^ HS DNA polymerase (Bioline, Boston, MA, USA) according to the manufacturer instructions. Amplification products were sequenced by Sanger sequencing at the Australian Genome Research Facility Ltd (http://www.agrf.org.au). All primer pairs amplified PCR products of expected length from both cvs. Gladius and Chinese Spring.

### Phylogenetic analyses

Phylogenetic analyses of the TaNAAT1/TaNAAT2 and TaDMAS1 protein sequences were conducted using PhyML algorithms (Geneious plugins) developed by [53]. An LG substitution model with four categories of distribution and 1000 bootstraps was performed on the aligned protein sequences respectively in Geneious Pro 8.1.7 software [54]. An empirical bootstrap proportion of branch topology ≥70% was considered indicative that the corresponding group was real with a probability ≥95 [55]. Published NAAT and DMAS1 protein sequences rice, barley and maize (S3 Table) were included in the phylogenetic analyses. A second putative NAAT protein in maize, ZmNAAT2, was identified following a BLAST analysis of OsNAAT1 amino acid sequence against the maize genome database (http://www.maizegdb.org/).

### Construction of cDNA libraries

Total RNA was extracted from 10 different tissues of bread wheat cv. Chinese Spring, the reference genome of the IWGSC, harvested at several stages of development including germination, seedling and maturity as described in [56]. Tissues were pooled from 7–10 plants per biological sample. Three independent biological samples were combined for each bulk of cDNA. The 10 cDNA tissue bulks used in our study were: mesocotyl and embryonic root (2 day old embryos); seedling root, crown and leaf collected 10–12 days after sowing (DAS); bracts, anthers, pistil (prior to anthesis); caryopsis and embryo collected 5 and 22 days after pollination (DAP) respectively.

Total RNA was extracted from root and shoot tissues of bread wheat cv. Gladius, a high-yielding Australian cultivar, as described in [20]. In brief, wheat seedlings were grown in full nutrient hydroponic solution for three weeks and then grown for an additional week in either Fe deficient or full nutrient solution. Tissue was harvested for RNA extraction at four time points (days 0, 1, 5 and 7 of the Fe deficient and control conditions) and cDNA was synthesized from three independent biological samples at each time point.

### Quantitative reverse transcription-PCR (qRT-PCR) analysis of *TaNAAT1*, *TaNAAT2* and *TaDMAS1* genes

Subgenome-specific qRT-PCR primer pairs were designed for each of the *TaNAAT1*, *TaNAAT2* and *TaDMAS1* genes using predicted CDS sequences and Primer3 software (Table 2) [57]. The specificity of each primer pair was confirmed through analysis of melt-curve and sequencing data. Primer efficiency was ≥97.83% for all primer pairs used in qRT-PCR analysis and was calculated using the formula 10^(-1/m)^ -1, where m corresponds to the slope of the standard curve generated using triplicate ten-fold serial dilutions (101–10^7^) of purified template for each primer pair. Optimisation of primer annealing temperature and efficiency as well as qRT-PCR analysis was performed at the Australian Centre for Plant Functional Genomics (ACPFG) Adelaide, Australia.

**Table 2:** Quantitative reverse transcription analysis of the *TaNAAT1*, *TaNAAT2* and *TaDMAS1* genes.

Wheat gene name	Forward primer sequence (5’-3’)	Reverse primer sequence (5’-3’)	Annealing temperature (°C)	PCR product length (bp)
***TaNAAT1-A***	CAGCAACCTTCGTCCAGGCT	ATCCTTCTGGCTTGTGAGGA	66	159
***TaNAAT1-B***	TTGGAGGAGATCCATGACG	TCCTTTCGAGACCATCTTCAA	60	154
***TaNAAT1-D***	AAAGGAAAACAAATACATTACATGC	CCGATTCTTCCTTTGCAAGC	66	102
***TaNAAT2-A***	GTCCAAGTCATGGATAGTTCCT	CAGACCGATAATCCCCTTGA	65	205
***TaNAAT2-B***	AATTGGAGGTTCGACATTTTG	CTTTCTTGCCACCTCTGCAA	60	179
***TaNAAT2-D***	CAGGCCAGGCTATCCAAAT	GCCAAATGGTCGTAGGAGTACA	63	198
***TaDMAS1-A***	CGTCCAGGGGCAAGAGTG	GAGCTCCTCGAGGGACTTGT	65	233
***TaDMAS1-B***	GGTGTGGCAGCAGAGGAA	CAGTGCGTGCCCTTGGCT	60	93
***TaDMAS1-D***	GAGGACTTCGTGCCCTTC	TGCAGAACTCCCTCAGCTTT	63	205
***TaActin***	GACAATGGAACCGGAATGGTC	GTGTGATGCCAGATTTTCTCCAT	60	236
***TaGAPDH***	TTCAACATCATTCCAAGCAGCA	CGTAACCCAAAATGCCCTTG	60	220
***TaCyclophilin***	CAAGCCGCTGCACTACAAGG	AGGGGACGGTGCAGATGAA	60	227
***TaELF***	CAGATTGGCAACGGCTACG	CGGACAGCAAAACGACCAAG	60	227

A three gene normalisation factor (3GNF) of house-keeping genes: cyclophilin (*TaCyc*), actin (*TaActin*) and elongation factor 1-alpha (*TaEFA*) for cv. Chinese Spring and *TaCyc*, *TaActin* and glyceraldehyde 3-phosphate dehydrogenase (*TaGAPDH*) for cv. Gladius was used to normalise qRT-PCR expression data as previously described [56, 58]. Expression data of cv. Gladius was normalised independently for shoot and root tissues (S3 Fig). A 3GNF for each of the 10 cv. Chinese Spring tissues was generated using a global standardisation factor (SF). The SF was calculated according to the following formula: SF=10^(∑log10(gm))n where SF = standardisation factor, gm = geometric mean of expression of 3 housekeeping genes within each tissue analysed and n = number of tissues analysed.

### Statistical analyses

Statistically significant differences in cv. Gladius cDNA expression levels were determined by Student’s t-test comparing the effect of treatment (+Fe and −Fe) at each time point (days 0, 1, 5 and 7) using Minitab 17.0 software (https://www.minitab.com/en-us/). Significant differences in expression levels were determined using Tukey’s test (p ≤0.05).

## Supporting information

S1 Fig Relative expression of the *TaNAAT1*, *TaNAAT2* and *TaDMAS1* genes in shoot and root tissues of bread wheat cv. Gladius under iron-sufficient/deficient conditions. Relative expression of all (**A**) *TaNAAT1*, (**B**) *TaNAAT2* and (**C**) *TaDMAS1* genes is presented at four time points of the 7 day treatment: days 0 (experiment start), 1, 5 and 7 of Fe-sufficient (+Fe, solid line) or Fe-deficient (-Fe, dashed line) conditions. Units of the y-axis indicate copies of mRNA per μl of cDNA. The error bars indicate standard error of the mean of three biological replicates for each of three genes (n = 9). Asterisks indicate significant differences for the effect of condition (+Fe and −Fe) at each time point (two-sample Student’s t-test assuming equal variance; * = p value ≤0.05; ** = p value ≤0.01; *** = p value ≤0.001).
(DOCX)

S2 Fig Annotation of cis-acting elements within the 1kb promoter regions of the TaNAAT1, TaNAAT2 and TaDMAS1 genes. Bold numbers indicate upstream positions of the TATA-box and the IDE1 and IDE2-like elements. Positions of the TaNAAT2-A and TaNAAT2-D TATA-box were validated using a wheat EST (GenBank: CA677231.1).
(DOCX)

S3 Fig Expression of three housekeeping genes in bread wheat shoot and root tissues prior to normalization. The expression of *TaActin* (black), *TaGAPDH* (grey) and *TaCyclophilin* (white) genes in bread wheat cv. Gladius (**A**) shoot and (**B**) root tissues. Tissue was harvested across 4 time points corresponding to days 0 (experiment start), 1, 5 and 7 from plants grown under Fe sufficient (+Fe, solid line) or Fe deficient (-Fe, dashed line) conditions. Units of the y-axis indicate copies of mRNA per μl of cDNA. The error bars indicate standard deviation of the mean of three technical replicates.
(DOCX)

S1 Table Percentage of sequence identity between the *TaNAAT* genes and proteins in bread wheat. The nucleotide identity of *TaNAAT* full coding sequences, first exon and exons 2–7 and amino acid identity for the TaNAAT full protein sequences are provided.
(DOCX)

S2 Table Percentage of sequence identity between the *TaDMAS1* genes and proteins in bread wheat. The nucleotide identity of *TaDMAS1* full coding sequences and amino acid identity for the TaDMAS full protein sequences are provided.
(DOCX)

S3 Table The rice, barley and maize proteins used in protein sequence and phylogenetic analyses. The gene name utilised in the manuscript and the corresponding accessions numbers from [11–13] are provided.
(DOCX)

S4 Table Expression data of the *TaNAAT1*, *TaNAAT2* and *TaDMAS1* genes in 10 different tissues and developmental stages of bread wheat cv. Chinese Spring. (XLSX)

S5 Table Expression data of the *TaNAAT1*, *TaNAAT2* and *TaDMAS1* genes in shoot and root tissues of bread wheat cv. Gladius under iron-sufficient/deficient conditions. (XLSX)
