# Supplementary material for: Characterisation of the nicotianamine aminotransferase and deoxymugineic acid synthase genes essential to Strategy II iron uptake in bread wheat (Triticum aestivum L.)
Source: PLoS One. 2017 May 5;12(5):e0177061. doi: 10.1371/journal.pone.0177061 (PMC5419654; doi:10.1371/journal.pone.0177061)
Supplement: S1 Fig — Relative expression of all (A) TaNAAT1, (B) TaNAAT2 and (C) TaDMAS1 genes is presented at four time points of the 7 day treatment: days 0 (experiment start), 1, 5 and 7 of Fe-sufficient (+Fe, solid line) or Fe-deficient (-Fe, dashed line) conditions. Units of the y-axis indicate copies of mRNA per μl of cDNA. The error bars indicate standard error of the mean of three biological replicates for each of three genes (n = 9). Asterisks indicate significant differences for the effect of condition (+Fe and −Fe) at each time point (two-sample Student’s t-test assuming equal variance; * = p value ≤0.05; ** = p value ≤0.01; *** = p value ≤0.001). (DOCX) [file pone.0177061.s001.docx]

**Figure S1** Relative expression of the *TaNAAT1*, *TaNAAT2* and *TaDMAS1* genes in shoot and root tissues of bread wheat cv. Gladius under iron-sufficient/deficient conditions. Relative expression of all (**A**) *TaNAAT1,* (**B**) *TaNAAT2* and (**C**) *TaDMAS1* genes is presented at four time points of the 7 day treatment: days 0 (experiment start), 1, 5 and 7 of Fe-sufficient (+Fe, solid line) or Fe-deficient (-Fe, dashed line) conditions. Units of the y-axis indicate copies of mRNA per µl of cDNA. The error bars indicate standard error of the mean of three biological replicates for each of three genes (n=9). Asterisks indicate significant differences for the effect of condition (+Fe and –Fe) at each time point (two-sample Student’s t-test assuming equal variance; * = p value ≤0.05; ** = p value ≤0.01; *** = p value ≤0.001).
