# Supplementary material for: Characterisation of the nicotianamine aminotransferase and deoxymugineic acid synthase genes essential to Strategy II iron uptake in bread wheat (Triticum aestivum L.)
Source: PLoS One. 2017 May 5;12(5):e0177061. doi: 10.1371/journal.pone.0177061 (PMC5419654; doi:10.1371/journal.pone.0177061)
Supplement: S2 Fig — Bold numbers indicate upstream positions of the TATA-box and the IDE1 and IDE2-like elements. Positions of the TaNAAT2-A and TaNAAT2-D TATA-box were validated using a wheat EST (GenBank: CA677231.1). (DOCX) [file pone.0177061.s002.docx]

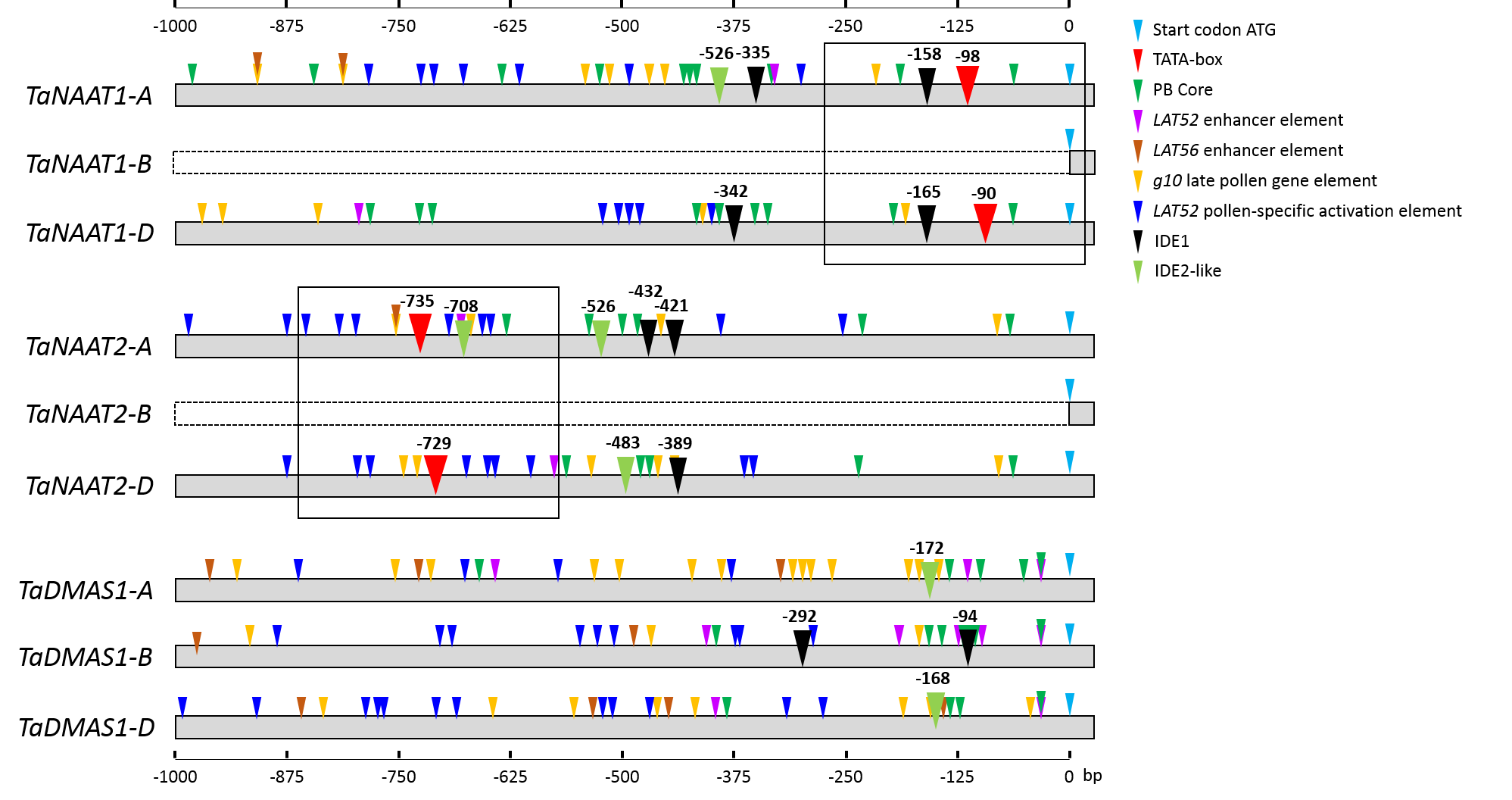


**S2 Fig Annotation of cis-acting elements within the 1kb promoter regions of the *TaNAAT1*, *TaNAAT2* and *TaDMAS1* genes.** Bold numbers indicate upstream positions of the TATA-box and the IDE1 and IDE2-like elements. Positions of the TaNAAT2-A and TaNAAT2-D TATA-box were validated using a wheat EST (GenBank: CA677231.1).
