# Supplementary material for: Characterisation of the nicotianamine aminotransferase and deoxymugineic acid synthase genes essential to Strategy II iron uptake in bread wheat (Triticum aestivum L.)
Source: PLoS One. 2017 May 5;12(5):e0177061. doi: 10.1371/journal.pone.0177061 (PMC5419654; doi:10.1371/journal.pone.0177061)
Supplement: S3 Fig — The expression of TaActin (black), TaGAPDH (grey) and TaCyclophilin (white) genes in bread wheat cv. Gladius (A) shoot and (B) root tissues. Tissue was harvested across 4 time points corresponding to days 0 (experiment start), 1, 5 and 7 from plants grown under Fe sufficient (+Fe, solid line) or Fe deficient (-Fe, dashed line) conditions. Units of the y-axis indicate copies of mRNA per μl of cDNA. The error bars indicate standard deviation of the mean of three technical replicates. (DOCX) [file pone.0177061.s003.docx]

**

**S3 Fig Expression of three housekeeping genes in bread wheat shoot and root tissues prior to normalization**. The expression of *TaActin* (black), *TaGAPDH* (grey) and *TaCyclophilin* (white) genes in bread wheat cv. Gladius (**A**) shoot and (**B**) root tissues. Tissue was harvested across 4 time points corresponding to days 0 (experiment start), 1, 5 and 7 from plants grown under Fe sufficient (+Fe, solid line) or Fe deficient (-Fe, dashed line) conditions. Units of the y-axis indicate copies of mRNA per µl of cDNA. The error bars indicate standard deviation of the mean of three technical replicates.
