# Supplementary material for: Characterisation of the nicotianamine aminotransferase and deoxymugineic acid synthase genes essential to Strategy II iron uptake in bread wheat (Triticum aestivum L.)
Source: PLoS One. 2017 May 5;12(5):e0177061. doi: 10.1371/journal.pone.0177061 (PMC5419654; doi:10.1371/journal.pone.0177061)
Supplement: S1 Table — The nucleotide identity of TaNAAT full coding sequences, first exon and exons 2–7 and amino acid identity for the TaNAAT full protein sequences are provided. (DOCX) [file pone.0177061.s004.docx]

**Table S1** Percentage of sequence identity between the TaNAAT genes and proteins in bread wheat. The nucleotide identity of TaNAAT full coding sequences, first exon and exons 2-7 and amino acid identity for the TaNAAT full protein sequences are provided.

Full coding sequence

|  | *TaNAAT1-A* | *TaNAAT1-B* | *TaNAAT1-D* | *TaNAAT2-A* | *TaNAAT2-B* | *TaNAAT2-D* |
| --- | --- | --- | --- | --- | --- | --- |
| *TaNAAT1-A* | 100 |  |  |  |  |  |
| *TaNAAT1-B* | 90.95 | 100 |  |  |  |  |
| *TaNAAT1-D* | 89.00 | 93.67 | 100 |  |  |  |
| *TaNAAT2-A* | 87.85 | 87.10 | 85.90 | 100 |  |  |
| *TaNAAT2-B* | 81.88 | 86.02 | 87.26 | 88.80 | 100 |  |
| *TaNAAT2-D* | 84.19 | 88.49 | 89.94 | 91.70 | 94.11 | 100 |

First exon of coding sequence

|  | *TaNAAT1-A* | *TaNAAT1-B* | *TaNAAT1-D* | *TaNAAT2-A* | *TaNAAT2-B* | *TaNAAT2-D* |
| --- | --- | --- | --- | --- | --- | --- |
| *TaNAAT1-A* | 100 |  |  |  |  |  |
| *TaNAAT1-B* | 80.22 | 100 |  |  |  |  |
| *TaNAAT1-D* | 74.95 | 87.40 | 100 |  |  |  |
| *TaNAAT2-A* | 78.01 | 78.34 | 73.86 | 100 |  |  |
| *TaNAAT2-B* | 62.38 | 75.42 | 78.94 | 74.86 | 100 |  |
| *TaNAAT2-D* | 67.26 | 80.56 | 84.80 | 78.24 | 90.50 | 100 |

Second to seventh exon of coding sequence

|  | *TaNAAT1-A* | *TaNAAT1-B* | *TaNAAT1-D* | *TaNAAT2-A* | *TaNAAT2-B* | *TaNAAT2-D* |
| --- | --- | --- | --- | --- | --- | --- |
| *TaNAAT1-A* | 100 |  |  |  |  |  |
| *TaNAAT1-B* | 95.73 | 100 |  |  |  |  |
| *TaNAAT1-D* | 95.73 | 96.73 | 100 |  |  |  |
| *TaNAAT2-A* | 92.76 | 92.36 | 92.36 | 100 |  |  |
| *TaNAAT2-B* | 92.26 | 91.67 | 91.77 | 96.13 | 100 |  |
| *TaNAAT2-D* | 92.66 | 92.46 | 92.56 | 98.31 | 96.03 | 100 |

Full protein sequence

|  | TaNAAT1-A | TaNAAT1-B | TaNAAT1-D | TaNAAT2-A | TaNAAT2-B | TaNAAT2-D |
| --- | --- | --- | --- | --- | --- | --- |
| TaNAAT1-A | 100 |  |  |  |  |  |
| TaNAAT1-B | 92.16 | 100 |  |  |  |  |
| TaNAAT1-D | 89.72 | 93.79 | 100 |  |  |  |
| TaNAAT2-A | 88.94 | 88.80 | 85.54 | 100 |  |  |
| TaNAAT2-B | 82.30 | 86.77 | 87.43 | 86.96 | 100 |  |
| TaNAAT2-D | 84.69 | 89.07 | 90.12 | 90.85 | 93.00 | 100 |
