# Supplementary material for: Characterisation of the nicotianamine aminotransferase and deoxymugineic acid synthase genes essential to Strategy II iron uptake in bread wheat (Triticum aestivum L.)
Source: PLoS One. 2017 May 5;12(5):e0177061. doi: 10.1371/journal.pone.0177061 (PMC5419654; doi:10.1371/journal.pone.0177061)
Supplement: S2 Table — The nucleotide identity of TaDMAS1 full coding sequences and amino acid identity for the TaDMAS full protein sequences are provided. (DOCX) [file pone.0177061.s005.docx]

**Table S2** Percentage of sequence identity between the TaDMAS1 genes and proteins in bread wheat. The nucleotide identity of TaDMAS1 full coding sequences and amino acid identity for the TaDMAS full protein sequences are provided.

Full coding sequence

|  | TaDMAS1-A | TaDMAS1-B | TaDMAS1-D |
| --- | --- | --- | --- |
| TaDMAS1-A | 100 |  |  |
| TaDMAS1-B | 97.9 | 100 |  |
| TaDMAS1-D | 97.8 | 98.2 | 100 |

Full protein sequence

|  | TaDMAS1-A | TaDMAS1-B | TaDMAS1-D |
| --- | --- | --- | --- |
| TaDMAS1-A | 100 |  |  |
| TaDMAS1-B | 98.4 | 100 |  |
| TaDMAS1-D | 97.8 | 98.1 | 100 |
