# Supplementary material for: Characterisation of the nicotianamine aminotransferase and deoxymugineic acid synthase genes essential to Strategy II iron uptake in bread wheat (Triticum aestivum L.)
Source: PLoS One. 2017 May 5;12(5):e0177061. doi: 10.1371/journal.pone.0177061 (PMC5419654; doi:10.1371/journal.pone.0177061)
Supplement: S3 Table — The gene name utilised in the manuscript and the corresponding accessions numbers from [11–13] are provided. (DOCX) [file pone.0177061.s006.docx]

**Table S3** The rice, barley and maize proteins used in protein sequence and phylogenetic analyses. The gene name utilised in the manuscript and the corresponding accessions numbers from Takahashi et al., (1999), Bashir et al., (2006) and Inoue et al., (2008) are provided.

| **Protein name** | **GenBank ID** |
| --- | --- |
| OsNAAT1 | LOC_Os02g20360 |
| OsNAAT2 | LOC_Os02g19970 |
| OsNAAT3 | LOC_Os02g19924 |
| OsNAAT4 | LOC_Os06g23684 |
| OsNAAT5 | LOC_Os11g35040 |
| OsNAAT6 | LOC_Os11g42510 |
| OsDMAS1 | LOC_Os03g13390 |
| HvNAATA | D88273 |
| HvNAATB | AB005788 |
| HvDMAS1 | AB269907 |
| ZmNAAT1 | GRMZM2G096958 |
| ZmNAAT2 | GRMZM2G412604 |
| ZmDMAS1 | BAF03164 |
